# Abnormal serum phosphorus was associated with the outcome of community-acquired pneumonia

**DOI:** 10.3389/fnut.2025.1435805

**Published:** 2025-06-04

**Authors:** Si-Qiong Wang, Cui-Hua Ma, Chun-Ming Ma, Rui Wang

**Affiliations:** First Hospital of Qinhuangdao, Qinhuangdao, China

**Keywords:** serum phosphorus, community-acquired pneumonia, outcome, abnormal serum phosphorus, outcome of CAP

## Abstract

**Objective:**

The present study aimed to explore the relationship between serum phosphorus levels and outcomes in patients with community-acquired pneumonia (CAP).

**Methods:**

This research was a retrospective, single-center study conducted on adult patients who were hospitalized with CAP at The First Hospital of Qinhuangdao City, Hebei Province, China, between January 2015 and December 2018. The primary outcome was in-hospital mortality. Participants were categorized into four groups: the normal serum phosphorus group (0.81–1.45 mmol/L), the hypophosphatemia group (Grade 1, 0.48–0.80 mmol/L), the hypophosphatemia group (Grade 2, <0.48 mmol/L), and the hyperphosphatemia group (>1.45 mmol/L).

**Results:**

This study included 1,936 CAP inpatients. The in-hospital mortality rates were 2.5, 4.4, 11.1, and 18.0% in the normal phosphorus group, the hypophosphatemia groups (Grades 1 and 2), and the hyperphosphatemia group, respectively. In the univariate logistic regression analysis, the in-hospital mortality rates for the hypophosphatemia (Grade 2) and hyperphosphatemia groups were 4.892 (95% CI: 1.410–16.969, *p* = 0.012) and 8.572 times (95% CI: 4.912–14.960, *p* < 0.001) higher, respectively, compared to the normal phosphorus group. After adjusting for confounding factors, hypophosphatemia (Grade 2) (OR = 3.715, 95% CI: 1.013–13.633, *p* = 0.048) and hyperphosphatemia (OR = 5.221, 95% CI: 2.747–9.924, *p* < 0.001) were identified as independent correlative factors associated with in-hospital mortality.

**Conclusion:**

Hyperphosphatemia and severe hypophosphatemia upon admission were associated with increased in-hospital mortality in CAP inpatients.

## Introduction

Community-acquired pneumonia (CAP) is one of the most common chronic respiratory diseases. The incidence rate of CAP ranges from 1.7/1,000 to 9.6/1,000 person-years (1–5). CAP is associated with high rates of hospitalization, significant healthcare costs, and prolonged hospital stays. A retrospective survey showed that there were 4,614,108 cases of hospitalization between 2010 and 2014 in New York, United States, of which 6.2% were due to pneumonia. More than half of these pneumonia cases were classified as community-acquired pneumonia (CAP) (6). A retrospective cohort study in the United States showed that the average length of hospital stay for CAP patients was 5.7 days and the average hospital cost was $17,736 (7). According to data from the Health Insurance Review and Assessment Service of Korea, the annual incidence of hospitalized cases of CAP was 626 per 100,000 people from 2009 to 2013, and the average medical cost per hospitalization was $1,851 (8). More importantly, hospitalized CAP cases were associated with significant mortality. Short-term mortality (in-hospital and 30-day mortality) for hospitalized patients ranged from 4–18% (9).

Phosphorus is an essential nutrient in the body. The primary function of phosphorus is related to the formation of bones and teeth. Approximately 85% of the body’s phosphorus is stored in the bones and teeth, while the remaining 15% is distributed throughout the blood and soft tissues. Serum phosphorus also plays an important role in lung health. Several studies have found that abnormal serum phosphorus levels are associated with an increased mortality rate in CAP patients (10, 11). However, not all observational studies support the link between serum phosphorus levels and outcomes in CAP patients (12). Therefore, the relationship between serum phosphorus levels and CAP should be explored in different populations.

Based on previous studies, we considered whether the association between abnormal serum phosphorus and community-acquired pneumonia differs among various populations in China. Therefore, we investigated the relationship between serum phosphorus levels and the severity and outcomes of community-acquired pneumonia in this study.

## Materials and methods

### Participants

We conducted a retrospective, single-center study involving inpatients at The First Hospital of Qinhuangdao, admitted between January 2015 and December 2018. The inclusion criteria were as follows: (1) patients who were admitted to the hospital due to CAP and (2) patients aged 18 years or older. The exclusion criteria were as follows: (1) patients who had no information regarding serum phosphorus levels, (2) patients with parathyroid diseases, renal tubular acidosis, and diabetic ketoacidosis, (3) patients with chronic obstructive pulmonary disease, lung cancer and asthma, and (4) patients lacking essential covariate data, such as sex, age, ethnicity, CURB-65 score, albumin levels, and the estimated glomerular filtration rate (eGFR). The ethics committee of The First Hospital of Qinhuangdao approved this study.

**Figure 1:**
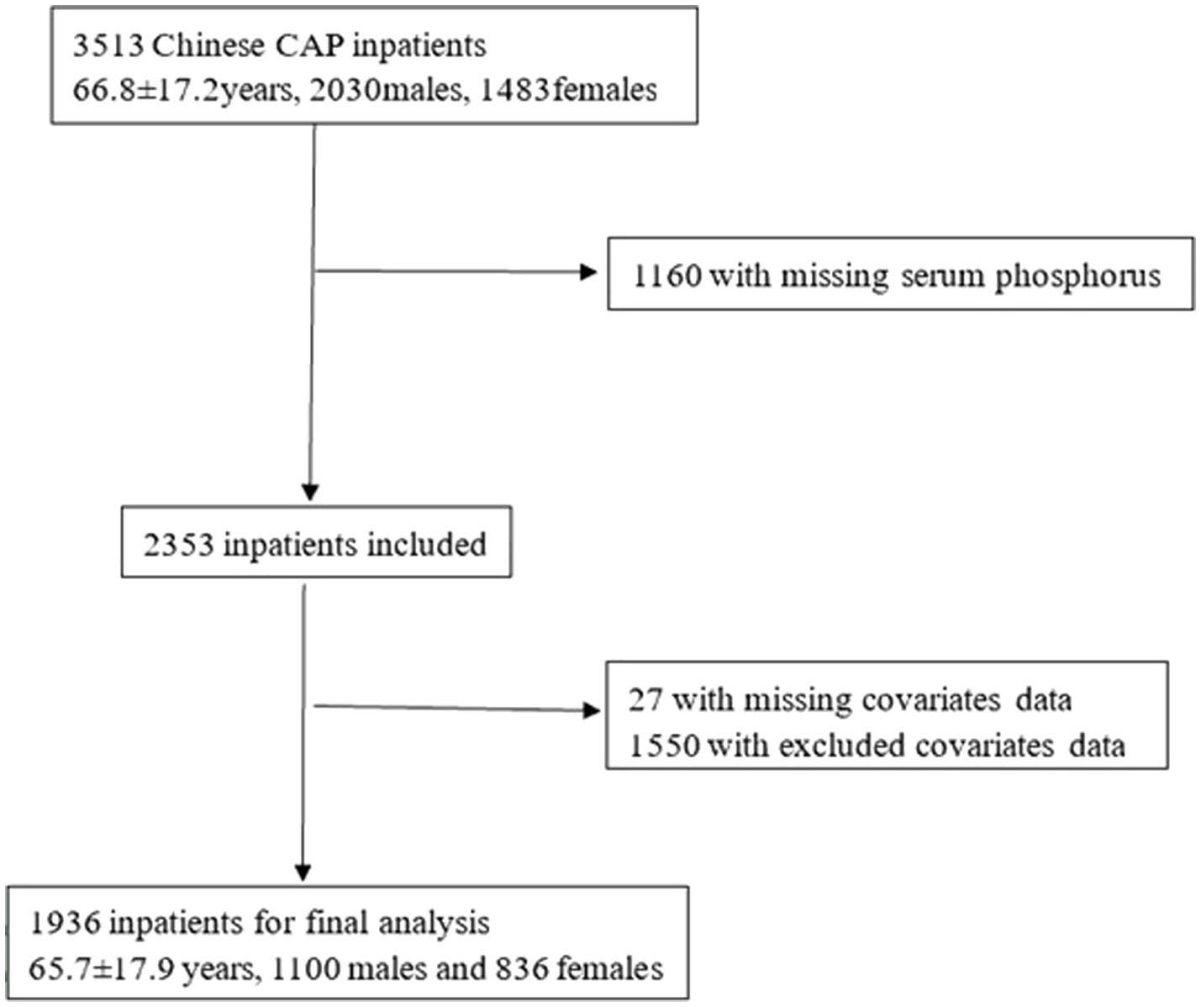
The flow chart of Chinese CAP inpatients.

A total of 3,513 CAP inpatients were initially identified in 2 cycles. We excluded inpatients with missing serum phosphorus data (*n* = 1,160). After further excluding inpatients with missing covariate data (*n* = 27) and those with parathyroid diseases, renal tubular acidosis, diabetic ketoacidosis, chronic obstructive pulmonary disease, lung cancer, asthma, or unavailable data (*n* = 1,550), a total of 1,936 inpatients were enrolled in the study (Figure 1).

### Data collection

The hospital information system provided the first set of data upon admission. In other words, all data included in this study were collected at the first measurement within 24 h of admission. Sociodemographic variables included sex, age, and ethnicity. Clinical data included diagnoses of diabetes, coronary heart disease (CHD), heart failure, and cerebrovascular disease. Physical examinations assessed mental status, respiratory rate, and blood pressure. Laboratory data included levels of urea, creatinine, albumin, and phosphorus. The eGFR was calculated using the following formula: (eGFR) = 175 × creatinine (mg/dL) − 1.234 × age (year) − 0.179 (×0.79 if female) (13).

The CURB-65 score [confusion, urea>7 mmol/L, respiratory rate ≥30/min, blood pressure (systolic blood pressure <90 mmHg or diastolic blood pressure ≤60 mmHg), and age ≥65 years] was calculated. Patients with a CURB-65 score of 0–1 were classified as the low-risk group, those with a score of 2 as the intermediate-risk group, and those with a score of 3–5 as the high-risk group (14).

### Definition of the exposure variable

Normal serum phosphorus levels were defined as 0.81–1.45 mmol/L (2.5–4.5 mg/dL) (11). Hypophosphatemia was defined as serum phosphorus concentrations below 0.81 mmol/L, while hyperphosphatemia was defined as serum phosphorus concentrations above 1.45 mmol/L. Participants in the study were categorized into four groups: normal serum phosphorus group (0.81–1.45 mmol/L), hypophosphatemia group (Grade 1, 0.48–0.80 mmol/L), hypophosphatemia group (Grade 2, <0.48 mmol/L), and hyperphosphatemia group (>1.45 mmol/L).

### Outcome

The primary outcome was in-hospital mortality. Data on respiratory failure, mechanical ventilation, septic shock, and intensive care unit (ICU) admission were also collected.

### Statistical analyses

Statistical analyses were performed using SPSS 24.0 software (SPSS Inc., Chicago, IL). A *p*-value of <0.05 was considered statistically significant. Numerical variables were presented as mean ± standard deviation. Analysis of variance (ANOVA) was employed to make comparisons between the groups. Chi-squared tests were performed to analyze categorical data, which were expressed as numbers and percentages of abnormal cases (%). Logistic regression analysis was performed to model the relationship between serum phosphorus levels and the outcome of CAP in patients. Model 1 involved univariate logistic regression with the serum phosphorus group as the predictor variable. Model 2 involved multiple logistic regression, adjusted for sex (female = 0, male = 1), age (<65 years = 0, ≥65 years = 1), ethnicity (Han = 0, other = 1), CURB-65 score (low risk = 0, intermediate risk = 1, high risk = 2), albumin levels (≥35 g/L = 0, <35 g/L = 1), eGFR (≥90 mL*min^−1^*(1.73m^2^)^−1^ = 0, <90 mL*min^−1^*(1.73m^2^)^−1^ = 1), diabetes (no = 0, yes = 1), CHD (no = 0, yes = 1), heart failure (no = 0, yes = 1), and cerebrovascular disease (no = 0, yes = 1). The analysis was also stratified by confounding factors. Based on the logistic regression models, restricted cubic spline (RCS) regression was performed to explore the non-linear relationship. We also examined the non-linear relationship between serum phosphorus levels as a continuous variable and the outcome of CAP, using a restricted cubic spline in the fully adjusted model, and confirmed the presence of an inflection point if a non-linear relationship was identified.

## Results

This study enrolled 1,936 patients with CAP (1,100 male and 836 female patients), with a mean age of 65.7 ± 17.9 years. Of these patients, 323 [16.7%, Grade 1: 296 patients (15.3%), Grade 2: 27 patients (1.4%)] had hypophosphatemia and 128 (6.6%) had hyperphosphatemia upon admission.

Table 1 shows the clinical characteristics of patients with different levels of serum phosphorus. The prevalence of male participants was higher in the hypophosphatemia (Grade 1) group compared to the normal phosphorus group (*p* < 0.05). Patients in the two hypophosphatemia groups (Grades 1 and 2) were older than those in the normal phosphorus group (*p* < 0.05). The prevalence of patients with Han ethnicity was similar across the four groups (*p* > 0.05). The CURB-65 scores were higher in patients in the three abnormal phosphorus groups compared to those in the normal phosphorus group (*p* < 0.05). Albumin levels were lower in patients in the three abnormal phosphorus groups compared to those in the normal phosphorus group (*p* < 0.05). The eGFR was lower in patients with hyperphosphatemia than in patients with normal phosphorus levels (*p* < 0.05). The prevalence of diabetes was similar across the four groups (*p* > 0.05). However, the prevalence of CHD was higher in hypophosphatemia (Grade 1) and hyperphosphatemia groups compared to the normal phosphorus group (*p* < 0.05). The prevalence of heart failure was higher in the hyperphosphatemia group than in the normal phosphorus group (*p* < 0.05). The prevalence of cerebrovascular disease was higher in two hypophosphatemia groups (Grades 1 and 2) compared to the normal phosphorus group (*p* < 0.05).

**Table 1:** The characteristics of community acquired pneumonia inpatients with different levels of serum phosphorus.

Variables	Serum phosphorus (mmol/L)				ALL (*N* = 1,936)	*F* or *χ*^2^	*p*
	0.81–1.45 (*N* = 1,485)	0.48–0.80 (*N* = 296)	<0.48 (*N* = 27)	>1.45 (*N* = 128)			
Sex [males *n* (%)]	804 (54.1)	208 (70.3)^a^	16 (59.3)	72 (56.3)	1,100 (56.8)	26.251	<0.001
Age (years)	64.6 ± 18.0	70.3 ± 16.1^a^	73.3 ± 16.5^a^	65.9 ± 18.2	65.7 ± 17.9	10.034	<0.001
Ethnicity [Han *n* (%)]	1,428 (96.2)	284 (95.9)	27 (100.0)	124 (96.9)	1,836 (96.2)	1.289	0.732
CURB-65 score	1.05 ± 0.96	1.31 ± 1.02^a^	1.67 ± 1.24^a^	1.91 ± 1.24^a^	1.15 ± 1.01	35.946	<0.001
Albumin (g/L)	35.7 ± 5.4	33.3 ± 5.2^a^	32.6 ± 4.8^a^	33.8 ± 6.5^a^	35.1 ± 5.5	21.002	<0.001
eGFR [mL*min^−1^*(1.73 m^2^)^−1^]	114.4 ± 49.1	118.0 ± 106.0	123.3 ± 110.1	67.9 ± 69.2^a^	112.0 ± 64.7	22.394	<0.001
Diabetes [*n* (%)]	313 (21.1)	68 (23.0)	5 (18.5)	33 (25.8)	419 (21.6)	2.037	0.565
CHD [*n* (%)]	338 (22.8)	87 (29.4)^a^	10 (37.0)	48 (37.5)^a^	483 (24.9)	19.793	<0.001
Heart failure [*n* (%)]	245 (16.5)	40 (13.5)	5 (18.5)	48 (37.5)^a^	338 (17.5)	39.845	<0.001
Cerebrovascular disease [*n* (%)]	392 (26.4)	101 (34.1)^a^	12 (44.4)^a^	33 (25.8)	538 (27.8)	11.338	0.010
Shock [*n* (%)]	29 (2.0)	16 (5.4)	5 (18.5)	22 (17.2)	72 (3.7)	99.648	<0.001
Death [*n* (%)]	37 (2.5)	13 (4.4)	3 (11.1)	23 (18.0)	76 (3.9)	78.894	<0.001

eGFR, estimated glomerular filtration rate; CHD, coronary heart disease; SD, standard deviation.

a Compared with serum phosphorus 0.81–1.45 mmol/L, *p* < 0.05. Numerical data are expressed as mean ± SD.

The prevalence of respiratory failure was 12.7% in the normal phosphorus group, 21.6% in the hypophosphatemia group (Grade 1), 22.2% in the hypophosphatemia group (Grade 2), and 36.7% in the hyperphosphatemia group. The prevalence of mechanical ventilation was 7.4% in the normal phosphorus group, 9.8% in the hypophosphatemia group (Grade 1), 25.9% in the hypophosphatemia group (Grade 2), and 32.0% in the hyperphosphatemia group respectively. The prevalence of septic shock was 2.0% in the normal phosphorus group, 5.4% in the hypophosphatemia group (Grade 1), 18.5% in the hypophosphatemia group (Grade 2), and 17.2% in the hyperphosphatemia group respectively. The prevalence of ICU admissions were 9.8% in the normal phosphorus group, 16.2% in the hypophosphatemia group (Grade 1), 33.3% in the hypophosphatemia group (Grade 2), and 45.3% in the hyperphosphatemia group respectively. The prevalence of septic shock was 2.0, 5.4, 18.5, and 17.2%, respectively. The proportions of ICU admissions were 9.8, 16.2, 33.3, and 45.3%, respectively. A total of 76 patients died during their hospital stay, and the in-hospital mortality rate was 3.9%. The in-hospital mortality rates were 2.5% in the normal phosphorus group, 4.4% in the hypophosphatemia group (Grade 1), 11.1% in the hypophosphatemia group (Grade 2), and 18.0% in the hyperphosphatemia group (Tables 2–6).

**Table 2:** The relationship between serum phosphorus and respiratory failure in patients with community acquired pneumonia.

Serum phosphorus (mmol/L)	*N* (%)^a^	*n* (%)^b^	Model 1		Model 2	
			OR (95% CI)	*p*	OR (95% CI)	*p*
<0.48	27 (1.4)	6 (22.2)	1.959 (0.781–4.916)	0.152	1.256 (0.470–3.358)	0.649
0.48–0.80	296 (15.3)	64 (21.6)	1.892 (1.379–2.595)	<0.001	1.605 (1.138–2.263)	0.007
0.81–1.45	1,485 (76.7)	189 (12.7)	1		1	
>1.45	128 (6.6)	47 (36.7)	3.979 (2.693–5.879)	<0.001	2.342 (1.500–3.658)	<0.001

Model 1: univariate logistic regression analysis. Model 2: multiple logistic regression analysis, adjusted for sex (females = 0, males = 1), age (<65 years = 0, ≥65 years = 1), ethnicity (Han = 0, other = 1), CURB-65 score (low risk = 0, intermediate risk = 1, high risk = 2), albumin (≥35 g/L = 0, <35 g/L = 1), eGFR (≥90 mL*min^−1^*(1.73 m^2^)^−1^ = 0, <90 mL*min^−1^*(1.73 m^2^)^−1^ = 1), diabetes (no = 0, yes = 1), CHD (no = 0, yes = 1), heart failure (no = 0, yes = 1) and cerebrovascular disease (no = 0, yes = 1). eGFR, estimated glomerular filtration rate; CHD, coronary heart disease; OR, odds ratio; CI, confidence interval.

a *N* (%): the number of patients with different levels of serum phosphorus (proportion).

b *n* (%): the number of respiratory failure in patients with different levels of serum phosphorus (proportion).

**Table 3:** The relationship between serum phosphorus and mechanical ventilation in patients with community acquired pneumonia.

Serum phosphorus (mmol/L)	*N* (%)*	*n* (%)^#^	Model 1		Model 2	
			OR (95%CI)	*P*	OR (95%CI)	*P*
<0.48	27 (1.4)	7 (25.9)	4.375 (1.810~10.573)	0.001	3.248 (1.238~8.523)	0.017
0.48~0.80	296 (15.3)	29 (9.8)	1.358 (0.884~2.086)	0.163	1.101 (0.695~1.745)	0.681
0.81~1.45	1485 (76.7)	110 (7.4)	1		1	
>1.45	128 (6.6)	41 (32.0)	5.891 (3.874~8.957)	<0.001	3.184 (1.970~5.146)	<0.001

*N (%): the number of patients with different levels of serum phosphorus (proportion), ^#^*n* (%): the number of mechanical ventilation in patients with different levels of serum phosphorus (proportion). Model 1: univariate logistic regression analysis. Model 2: multiple logistic regression analysis, adjusted for sex (Females = 0, Males = 1), age (<65 years = 0, ≥65 years = 1), ethnicity (Han = 0, Other = 1), CURB-65 Score (Low risk = 0, Intermediate risk = 1, High risk = 2), albumin (≥35g/L = 0, <35g/L = 1), eGFR (≥90 ml*min^−1^*(1.73 m^2^)n^−1^ = 0, <90 ml*min^−1^*(1.73 m^2^)^−1^ = 1), diabetes (No = 0, Yes = 1), CHD (No = 0, Yes = 1), heart failure (No = 0, Yes = 1) and cerebrovascular disease (No = 0, Yes = 1). eGFR: estimated glomerular filtration rate; CHD: coronary heart disease; OR: odds ratio; CI: confidence interval.

**Table 4:** The relationship between serum phosphorus and septic shock in patients with community acquired pneumonia.

Serum phosphorus (mmol/L)	*N* (%)^a^	*n* (%)^b^	Model 1		Model 2	
			OR (95% CI)	*p*	OR (95% CI)	*p*
<0.48	27 (1.4)	5 (18.5)	11.411 (4.040–32.227)	<0.001	9.164 (2.844–29.527)	<0.001
0.48–0.80	296 (15.3)	16 (5.4)	2.869 (1.538–5.353)	0.001	2.381 (1.225–4.628)	0.011
0.81–1.45	1,485 (76.7)	29 (2.0)	1		1	
>1.45	128 (6.6)	22 (17.2)	10.420 (5.787–18.764)	<0.001	4.204 (2.169–8.146)	<0.001

a *N* (%): the number of patients with different levels of serum phosphorus (proportion).

b *n* (%): the number of septic shock in patients with different levels of serum phosphorus (proportion).

Model 1: univariate logistic regression analysis. Model 2: multiple logistic regression analysis, adjusted for sex (females = 0, males = 1), age (<65 years = 0, ≥65 years = 1), ethnicity (Han = 0, other = 1), CURB-65 score (low risk = 0, intermediate risk = 1, high risk = 2), albumin (≥35 g/L = 0, <35 g/L = 1), eGFR (≥90 mL*min^−1^*(1.73 m^2^)^−1^ = 0, <90 mL*min^−1^*(1.73 m^2^)^−1^ = 1), diabetes (no = 0, yes = 1), CHD (no = 0, yes = 1), heart failure (no = 0, yes = 1) and cerebrovascular disease (no = 0, yes = 1). eGFR, estimated glomerular filtration rate; CHD, coronary heart disease; OR, odds ratio; CI, confidence interval.

**Table 5:** The relationship between serum phosphorus and ICU admissions in patients with community acquired pneumonia.

Serum phosphorus (mmol/L)	*N* (%)^a^	*n* (%)^b^	Model 1		Model 2	
			OR (95% CI)	*p*	OR (95% CI)	*p*
<0.48	27 (1.4)	9 (33.3)	4.586 (2.023–10.393)	<0.001	3.494 (1.334–9.153)	0.011
0.48–0.80	296 (15.3)	48 (16.2)	1.775 (1.247–2.527)	0.001	1.531 (1.025–2.286)	0.038
0.81–1.45	1,485 (76.7)	146 (9.8)	1		1	
>1.45	128 (6.6)	58 (45.3)	7.599 (5.157–11.198)	<0.001	3.913 (2.472–6.196)	<0.001

a *N* (%): the number of patients with different levels of serum phosphorus (proportion).

b *n* (%): the number of ICU admissions in patients with different levels of serum phosphorus (proportion).

Model 1: univariate logistic regression analysis. Model 2: multiple logistic regression analysis, adjusted for sex (females = 0, males = 1), age (<65 years = 0, ≥65 years = 1), ethnicity (Han = 0, other = 1), CURB-65 score (low risk = 0, intermediate risk = 1, high risk = 2), albumin (≥35 g/L = 0, <35 g/L = 1), eGFR (≥90 mL*min^−1^*(1.73 m^2^)^−1^ = 0, <90 mL*min^−1^*(1.73 m^2^)^−1^ = 1), diabetes (no = 0, yes = 1), CHD (no = 0, yes = 1), heart failure (no = 0, yes = 1) and cerebrovascular disease (no = 0, yes = 1). ICU, intensive care unit; eGFR, estimated glomerular filtration rate; CHD, coronary heart disease; OR, odds ratio; CI, confidence interval.

**Table 6:** The relationship between serum phosphorus and in-hospital mortality in patients with community acquired pneumonia.

Serum phosphorus (mmol/L)	*N* (%)^a^	*n* (%)^b^	Model 1		Model 2	
			OR (95% CI)	*p*	OR (95% CI)	*p*
<0.48	27 (1.4)	3 (11.1)	4.892 (1.410–16.969)	0.012	3.715 (1.013–13.633)	0.048
0.48–0.80	296 (15.3)	13 (4.4)	1.798 (0.944–3.425)	0.075	1.470 (0.749–2.885)	0.263
0.81–1.45	1,485 (76.7)	37 (2.5)	1		1	
>1.45	128 (6.6)	23 (18.0)	8.572 (4.912–14.960)	<0.001	5.221 (2.747–9.924)	<0.001

Model 1: univariate logistic regression analysis. Model 2: multiple logistic regression analysis, adjusted for sex (females = 0, males = 1), age (<65 years = 0, ≥65 years = 1), ethnicity (Han = 0, other = 1), CURB-65 score (low risk = 0, intermediate risk = 1, high risk = 2), albumin (≥35 g/L = 0, <35 g/L = 1), eGFR (≥90 mL*min^−1^*(1.73 m^2^)^−1^ = 0, <90 mL*min^−1^*(1.73 m^2^)^−1^ = 1), diabetes (no = 0, yes = 1), CHD (no = 0, yes = 1), heart failure (no = 0, yes = 1) and cerebrovascular disease (no = 0, yes = 1). eGFR, estimated glomerular filtration rate; CHD, coronary heart disease; OR, odds ratio; CI, confidence interval.

a *N* (%): the number of patients with different levels of serum phosphorus (proportion).

b *n* (%): the number of death in patients with different levels of serum phosphorus (in-hospital mortality).

In the multiple logistic regression analysis, adjusted for sex, age, ethnicity, CURB-65 score, albumin levels, eGFR, diabetes, heart failure, and cerebrovascular disease, hypophosphatemia (Grade 1) (OR = 1.605, 95% CI: 1.138–2.263, *p* = 0.007) and hyperphosphatemia (OR = 2.342, 95% CI: 1.500–3.658, *p* < 0.001) were identified as independent correlative factors of respiratory failure (Table 2). In the multiple logistic regression analysis, adjusted for sex, age, ethnicity, CURB-65 score, albumin levels, eGFR, diabetes, heart failure, and cerebrovascular disease, hypophosphatemia (Grade 2) (OR = 3.248, 95% CI: 1.238–8.523, *p* = 0.017) and hyperphosphatemia (OR = 3.184, 95% CI: 1.970–5.146, *p* < 0.001) were identified as independent correlative factors of mechanical ventilation (Table 3). In the multiple logistic regression analysis, adjusted for sex, age, ethnicity, CURB-65 score, albumin levels, eGFR, diabetes, heart failure, and cerebrovascular disease, Grade 1 hypophosphatemia (OR = 2.381, 95% CI: 1.225–4.628, *p* = 0.011), Grade 2 hypophosphatemia (OR = 9.164, 95% CI: 2.844–29.527, *p* < 0.001), and hyperphosphatemia (OR = 4.204, 95% CI: 2.169–8.146, *p* < 0.001) were identified as independent correlative factors of septic shock (Table 4). In the multiple logistic regression analysis, adjusted for sex, age, ethnicity, CURB-65 score, albumin levels, eGFR, diabetes, heart failure, and cerebrovascular disease, Grade 1 hypophosphatemia (OR = 1.531, 95% CI: 1.025–2.286, *p* = 0.038), Grade 2 hypophosphatemia (OR = 3.494, 95% CI: 1.334–9.153, *p* = 0.011), and hyperphosphatemia (OR = 3.913, 95% CI: 2.472–6.196, *p* < 0.001) were identified as independent correlative factors of ICU admission (Table 5). In the univariate logistic regression analysis, the in-hospital mortality rates in hypophosphatemia (Grade 2) and hyperphosphatemia groups were 4.892 times (95% CI, 1.410–16.969, *p* = 0.012) and 8.572 times (95% CI, 4.912–14.960, *p* < 0.001) higher, respectively, compared to the normal phosphorus group (Table 6). In the multiple logistic regression analysis, adjusted for sex, age, ethnicity, CURB-65 score, albumin levels, eGFR, diabetes, heart failure, and cerebrovascular disease, hypophosphatemia (Grade 2) (OR = 3.715, 95% CI: 1.013–13.633, *p* = 0.048) and hyperphosphatemia (OR = 5.221, 95% CI: 2.747–9.924, *p* < 0.001) were identified as independent correlative factors of in-hospital mortality (Table 6).

The analysis was also stratified by confounding factors (Table 7). Except for patients of other ethnicities, hyperphosphatemia was correlated with an increased risk of in-hospital mortality in all stratified analyses (*p* < 0.05). Hypophosphatemia (Grade 1) was only associated with an increased risk of in-hospital mortality in patients without CHD or heart failure (*p* < 0.05). Five patients (0.6%) under the age of 65 years and 22 patients (1.9%) aged 65 years or older had hypophosphatemia (Grade 2). No patients under the age of 65 years died, whereas three patients (13.6%) aged 65 years or older died. Among the patients aged ≥ 65 years, those in the hypophosphatemia (Grade 2) group had an increased risk of in-hospital mortality compared to those in the normal phosphorus group, with an OR (95%CI) of 3.720 (1.050–13.181, *p* = 0.042).

**Table 7:** The relationship between serum phosphorus and in-hospital mortality in patients with community acquired pneumonia (stratified by confounding factors).

Stratified by		Serum phosphorus (mmol/L)	*N* (%)^a^	*n* (%)^b^	OR (95% CI)	*p*
Sex	Females (*N* = 836)	<0.48	11 (1.3)	0 (0.0)	NA	
		0.48–0.80	88 (10.5)	4 (4.5)	2.114 (0.686–6.519)	0.193
		0.81–1.45	681 (81.5)	15 (2.2)	1	
		>1.45	56 (6.7)	7 (12.5)	6.343 (2.471–16.285)	<0.001
	Males (*N* = 1,100)	<0.48	16 (1.5)	3 (18.8)	8.203 (2.180–30.859)	0.002
		0.48–0.80	208 (18.9)	9 (4.3)	1.608 (0.729–3.546)	0.239
		0.81–1.45	804 (73.1)	22 (2.7)	1	
		>1.45	72 (6.5)	16 (22.2)	10.156 (5.050–20.425)	<0.001
Age	<65 years (*N* = 799)	<0.48	5 (0.6)	0 (0.0)	NA	
		0.48–0.80	91 (11.4)	0 (0.0)	NA	
		0.81–1.45	650 (81.4)	3 (0.5)	1	
		>1.45	53 (6.6)	3 (5.7)	12.940 (2.546–65.778)	0.002
	≥65 years (*N* = 1,137)	<0.48	22 (1.9)	3 (13.6)	3.720 (1.050–13.181)	0.042
		0.48–0.80	205 (18.0)	13 (6.3)	1.595 (0.826–3.081)	0.164
		0.81–1.45	835 (73.4)	34 (4.1)	1	
		>1.45	75 (6.6)	20 (26.7)	8.567 (4.626–15.865)	<0.001
Ethnicity	Han (*N* = 1,863)	<0.48	27 (1.4)	3 (11.1)	4.975 (1.431–17.299)	0.012
		0.48–0.80	284 (15.2)	13 (4.6)	1.909 (0.997–3.656)	0.051
		0.81–1.45	1,428 (76.7)	35 (2.5)	1	
		>1.45	124 (6.7)	22 (17.7)	8.584 (4.855–15.178)	<0.001
	Other (*N* = 73)	<0.48	0 (0.0)	0 (0.0)	NA	
		0.48–0.80	12 (16.4)	0 (0.0)	NA	
		0.81–1.45	57 (78.1)	2 (3.5)	1	
		>1.45	4 (5.5)	1 (25.0)	9.167 (0.637–131.961)	0.103
CURB-65 Score	Low risk (*N* = 1,297)	<0.48	15 (1.2)	1 (6.7)	8.294 (0.983–69.945)	0.052
		0.48–0.80	184 (14.2)	2 (1.1)	1.276 (0.273–5.953)	0.756
		0.81–1.45	1,054 (81.3)	9 (0.9)	1	
		>1.45	44 (3.4)	2 (4.5)	5.529 (1.158–26.389)	0.032
	Intermediate risk (*N* = 444)	<0.48	5 (1.1)	1 (20.0)	5.375 (0.563–51.297)	0.144
		0.48–0.80	80 (18.0)	7 (8.8)	2.062 (0.803–5.292)	0.132
		0.81–1.45	315 (70.9)	14 (4.4)	1	
		>1.45	44 (9.9)	9 (20.5)	5.529 (2.231–13.702)	<0.001
	High risk (*N* = 195)	<0.48	7 (3.6)	1 (14.3)	1.214 (0.136–10.844)	0.862
		0.48–0.80	32 (16.4)	4 (12.5)	1.041 (0.318–3.412)	0.947
		0.81–1.45	116 (59.5)	14 (12.1)	1	
		>1.45	40 (20.5)	12 (30.0)	3.122 (1.299–7.506)	0.011
Albumin	≥35 g/L (*N* = 1,025)	<0.48	8 (0.8)	1 (12.5)	14.982 (1.647–136.292)	0.016
		0.48–0.80	110 (10.7)	1 (0.9)	0.962 (0.119–7.767)	0.971
		0.81–1.45	847 (82.6)	8 (0.9)	1	
		>1.45	60 (5.9)	8 (13.3)	16.135 (5.823–44.710)	<0.001
	<35 g/L (*N* = 911)	<0.48	19 (2.1)	2 (10.5)	2.471 (0.545–11.204)	0.241
		0.48–0.80	186 (20.4)	12 (6.5)	1.448 (0.724–2.898)	0.295
		0.81–1.45	638 (70.0)	29 (4.5)	1	
		>1.45	68 (7.5)	15 (22.1)	5.943 (3.000–11.774)	<0.001
eGFR	≥90 mL*min^−1^*(1.73 m^2^)^−1^ (*N* = 1,320)	<0.48	19 (1.4)	1 (5.3)	2.889 (0.368–22.705)	0.313
		0.48–0.80	199 (15.1)	8 (4.0)	2.178 (0.946–5.016)	0.067
		0.81–1.45	1,060 (80.3)	20 (1.9)	1	
		>1.45	42 (3.2)	3 (7.1)	4.000 (1.140–14.030)	0.030
	<90 mL*min^−1^*(1.73 m^2^)^−1^ (*N* = 616)	<0.48	8 (1.3)	2 (25.0)	8.000 (1.503–42.592)	0.015
		0.48–0.80	97 (15.7)	5 (5.2)	1.304 (0.469–3.626)	0.611
		0.81–1.45	425 (69.0)	17 (4.0)	1	
		>1.45	86 (14.0)	20 (23.3)	7.273 (3.623–14.600)	<0.001
Diabetes	No (*N* = 1,517)	<0.48	22 (1.5)	3 (13.6)	7.888 (2.181–28.532)	0.002
		0.48–0.80	228 (15.0)	9 (3.9)	2.053 (0.937–4.497)	0.072
		0.81–1.45	1,172 (77.3)	23 (2.0)	1	
		>1.45	95 (6.3)	14 (14.7)	8.634 (4.281–17.415)	<0.001
	Yes (*N* = 419)	<0.48	5 (1.2)	0 (0.0)	NA	
		0.48–0.80	68 (16.2)	4 (5.9)	1.335 (0.425–4.188)	0.621
		0.81–1.45	313 (74.7)	14 (4.5)	1	
		>1.45	33 (7.9)	9 (27.3)	8.009 (3.144–20.399)	<0.001
CHD	No (*N* = 1,453)	<0.48	17 (1.2)	1 (5.9)	5.508 (0.627–40.808)	0.128
		0.48–0.80	209 (14.4)	7 (3.3)	2.804 (1.118–7.034)	0.028
		0.81–1.45	1,147 (78.9)	14 (1.2)	1	
		>1.45	80 (5.5)	11 (13.8)	12.902 (5.647–29.477)	<0.001
	Yes (*N* = 483)	<0.48	10 (2.1)	2 (20.0)	3.424 (0.687–17.066)	0.133
		0.48–0.80	87 (18.0)	6 (6.9)	1.014 (0.400–2.574)	0.976
		0.81–1.45	338 (70.0)	23 (6.8)	1	
		>1.45	48 (9.9)	12 (25.0)	4.565 (2.096–9.944)	<0.001
Heart failure	No (*N* = 1,598)	<0.48	22 (1.4)	2 (9.1)	7.650 (1.648–35.504)	0.009
		0.48–0.80	256 (16.0)	9 (3.5)	2.787 (1.218–6.380)	0.015
		0.81–1.45	1,240 (77.6)	16 (1.3)	1	
		>1.45	80 (5.0)	12 (15.0)	13.500 (6.143–29.666)	<0.001
	Yes (*N* = 338)	<0.48	5 (1.5)	1 (20.0)	2.667 (0.285–24.961)	0.390
		0.48–0.80	40 (11.8)	4 (10.0)	1.185 (0.385–3.653)	0.767
		0.81–1.45	245 (72.5)	21 (8.6)	1	
		>1.45	48 (14.2)	11 (22.9)	3.171 (1.413–7.115)	0.005
Cerebrovascular disease	No (*N* = 1,398)	<0.48	15 (1.1)	0 (0.0)	NA	
		0.48–0.80	195 (13.9)	6 (3.1)	1.703 (0.675–4.297)	0.259
		0.81–1.45	1,093 (78.2)	20 (1.8)	1	
		>1.45	95 (6.8)	17 (17.9)	11.693 (5.887–23.224)	<0.001
	Yes (*N* = 538)	<0.48	12 (2.2)	3 (25.0)	7.353 (1.824–29.642)	0.005
		0.48–0.80	101 (18.8)	7 (6.9)	1.643 (0.662–4.076)	0.284
		0.81–1.45	392 (72.9)	17 (4.3)	1	
		>1.45	33 (6.1)	6 (18.2)	4.902 (1.787–13.450)	0.002

Univariate logistic regression analysis. eGFR, estimated glomerular filtration rate; CHD, coronary heart disease; OR, odds ratio; CI, confidence interval.

a *N* (%): the number of patients with different levels of serum phosphorus (proportion).

b *n* (%): the number of death in patients with different levels of serum phosphorus (in-hospital mortality).

**Figure 2:**
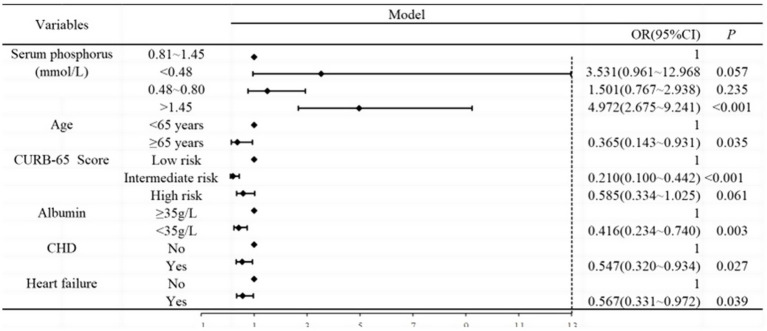
Forest plot analysis between serum phosphorus and outcome of CAP. Age, CURB-65 score, albumin, CHD, and heart failure were adjusted.

Based on Table 7, we analyzed the results of the multivariate logistic regression (Table 8), which revealed abnormal serum phosphorus levels (hyperphosphatemia), age (≥65 years), CURB-65 score (intermediate risk), albumin levels (<35 g/L), CHD, and heart failure as risk factors for mortality. These findings are also illustrated in Figure 2.

**Table 8:** The relationship between serum phosphorus and in-hospital mortality in patients with community acquired pneumonia (multivariate logistic regression was analyzed based on Table 7).

Variables		Model	
		OR (95% CI)	*p*
Serum phosphorus (mmol/L)	0.81–1.45	1	
	<0.48	3.531 (0.961–12.968)	0.057
	0.48–0.80	1.501 (0.767–2.938)	0.235
	>1.45	4.972 (2.675–9.241)	<0.001
Age	<65 years	1	
	≥65 years	0.365 (0.143–0.931)	0.035
CURB-65 score	Low risk	1	
	Intermediate risk	0.210 (0.100–0.442)	<0.001
	High risk	0.585 (0.334–1.025)	0.061
Albumin	≥35 g/L	1	
	<35 g/L	0.416 (0.234–0.740)	0.003
CHD	No	1	
	Yes	0.547 (0.320–0.934)	0.027
Heart failure	No	1	
	Yes	0.567 (0.331–0.972)	0.039

eGFR, estimated glomerular filtration rate; CHD, coronary heart disease; OR, odds ratio; CI, confidence interval.

**Figure 3:**
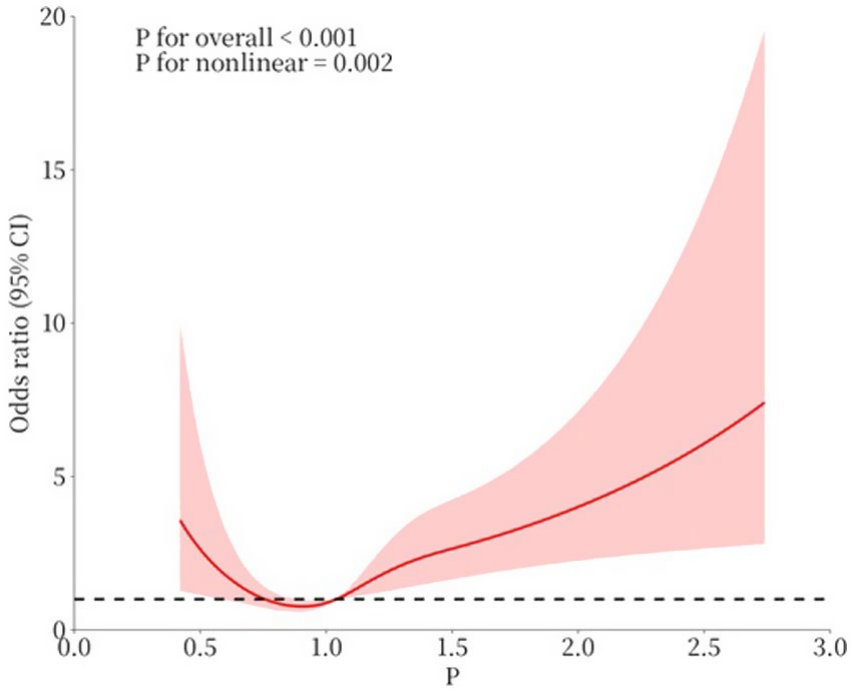
The association between serum levels of phosphate and outcome. (1) *p*: serum phosphate level (mmol/L). (2) Age, CURB-65 score, albumin, CHD, and heart failure were adjusted in the restricted cubic spline.

In the current study population, the relationship between serum phosphate levels and the outcome of CAP was evaluated using restricted cubic spline analysis, with a log-likelihood ratio test showing statistical significance at a *p*-value of <0.001 (Figure 3). The inflection point of the serum phosphate level was 1.04 mmol/L. Considering the significant association between serum phosphorus levels and the outcome of CAP, the inflection point was 1.04. We performed restricted cubic spline analyses based on the results in Table 8. As shown in Figure 3, the odds ratio (OR) exceeded 1 when serum phosphate levels reached 1.04 mmol/L.

## Discussion

Abnormal serum phosphorus levels were common in patients with CAP. According to serum phosphorus levels upon admission, approximately a quarter of CAP patients had abnormal serum phosphorus levels, especially hypophosphatemia. In our study, CAP patients who had abnormal serum phosphorus levels, whether hypophosphatemia or hyperphosphatemia, tended to have more severe or worse clinical outcomes.

Consistent with previous research (10, 11), hyperphosphatemia upon admission was linked to in-hospital mortality in patients with CAP. In the four groups, the in-hospital mortality rate was the highest in CAP patients who had hyperphosphatemia, reaching 18%. As expected, the patients in the hyperphosphatemia group had more severe clinical conditions. CAP patients who had hyperphosphatemia exhibited higher rates of respiratory failure, mechanical ventilation, septic shock, and ICU admission. The CURB-65 score is a widely used tool for evaluating the severity of CAP (14). The analysis was stratified by the severity of CAP according to the CURB-65 scores. Hyperphosphatemia was associated with increased in-hospital mortality in CAP patients, regardless of risk stratification. Decline in kidney function was found to be a major cause of hyperphosphatemia. The participants with a reduced eGFR had significantly higher serum phosphorus levels due to less phosphate excretion (15). High phosphorus levels were also associated with an increased risk of cardiovascular disease (16). After the stratification analysis, hyperphosphatemia was associated with increased in-hospital mortality in the patients with ≥90 mL*min^−1^*(1.73 m^2^)^−1^ and patients without CHD or heart failure. The results support the conclusion that the effect of hyperphosphatemia on the outcome of CAP is independent of these confounding factors.

Aging is characterized by gradual decline in organ function and an increased susceptibility to infections. Klotho, a transmembrane protein found in multiple organs, is a cofactor of FGF23 and FGF receptors. Mice that lack the klotho protein exhibit premature aging and develop hyperphosphatemia (17). In the study by Asenjo-Bueno et al. (18), it was found that aged mice exhibited hyperphosphatemia and impaired respiratory function. Older mice had higher serum phosphorus levels compared to younger mice. It has been reported that higher salivary phosphate concentrations are associated with higher inflammatory markers (17). Inflammation is closely related to aging (18).

The relationship between hypophosphatemia and the outcome of CAP remains controversial. Naffaa et al. (11) reported that abnormal serum phosphorus levels, including hypophosphatemia and hyperphosphatemia upon admission, were associated with increased mortality in patients hospitalized with CAP in Israel. However, Morimoto et al. (12) found that hypophosphatemia was not associated with prognosis in patients with CAP in Japan. The difference in findings can primarily be attributed to the definition of hypophosphatemia. In the study by Naffaa et al. (11), hypophosphatemia was defined as serum phosphorus levels below 2.5 mg/dL (0.81 mmol/L). Patients with hypophosphatemia were further divided into two groups: those with phosphorus levels ≤1.5 mg/dL (≤0.48 mmol/L) and those with phosphorus levels of 1.51–2.49 mg/dL (0.48–0.81 mmol/L). Increased mortality was only observed in patients with phosphorus levels ≤1.5 mg/dL (≤0.48 mmol/L). In the study by Morimoto et al. (12), hypophosphatemia was only defined as serum phosphorus levels below 2.0 mg/dL (0.65 mmol/L). In our study, increased mortality was only observed in patients with phosphorus levels <0.48 mmol/L (11.1%) but not in patients with phosphorus levels ranging between 0.48 and 0.81 mmol/L (4.4%). Severe hypophosphatemia (phosphorus <0.48 mmol/L) was associated with the clinical outcome in CAP inpatients, although it was rare, occurring in only 27 patients (1.4%). However, mild hypophosphatemia (0.48–0.80 mmol/L) was associated with the severity of CAP in our study. The prevalence of respiratory failure, septic shock, and ICU admission was higher in patients with mild hypophosphatemia.

Hypophosphatemia is commonly observed in older hospitalized patients, with prevalence rates ranging from 7 to 29% (19–21). Inadequate phosphate intake and increased phosphate excretion are two common causes of hypophosphatemia (22). Possible mechanisms of hypophosphatemia in patients with CAP include the following: (1) during the body’s emergency response, much of the phosphorus is utilized in energy metabolism and (2) in hypophosphatemia, the renal phosphorus threshold decreases, which reduces the absorption of phosphorus in the renal tubules and increases urinary phosphorus excretion (23). Limited intake of phosphorus, chronic disease, and medications may influence the level of serum phosphorus in elderly patients. In our study, the prevalence of hypophosphatemia was higher in patients aged ≥65 years (19.9%, Grade 1: 1.9%, Grade 2: 18.0%). In patients with hypophosphatemia, no deaths were reported among those aged <65 years. Hypophosphatemia (Grade 2) was associated with increased in-hospital mortality only in elderly CAP patients. This result is consistent with a study on hemodialysis patients (24).

There are several limitations to our study. First, abnormal serum phosphorus levels were defined based on the initial phosphorus level upon admission. Changes in serum phosphorus levels after admission were not analyzed in our study. Second, phosphorus intake and medications for chronic diseases were not evaluated in our study. Third, this was a single-center study, and only 27 patients had serum phosphorus levels <0.48 mmol/L. As a result, the findings of this study should be replicated in other populations.

## Conclusion

In conclusion, hyperphosphatemia upon admission was associated with increased in-hospital mortality rates in CAP inpatients. Hypophosphatemia was correlated with the severity of CAP, and only severe hypophosphatemia had an effect on the outcome of CAP.

## Data Availability

The raw data supporting the conclusions of this article will be made available by the authors, without undue reservation.
